# Translation and validation to the Arabic language version of the climate change anxiety scale (CCAS)

**DOI:** 10.1186/s12888-024-05956-0

**Published:** 2024-07-16

**Authors:** Feten Fekih-Romdhane, Diana Malaeb, Ecem Yakın, Fouad Sakr, Mariam Dabbous, Sami El Khatib, Sahar Obeid, Souheil Hallit

**Affiliations:** 1 The Tunisian Center of Early Intervention in Psychosis, Department of psychiatry Ibn Omrane, Razi Hospital, Manouba, 2010 Tunisia; 2 Faculty of Medicine of Tunis, Tunis El Manar University, Tunis, Tunisia; 3 College of Pharmacy, Gulf Medical University, Ajman, United Arab Emirates; 4 Centre d’Etudes Et de Recherches en Psychopathologie Et Psychologie de La Santé, Université de Toulouse-Jean Jaurès, UT2J, 5 Allées Antonio Machado, Toulouse, 31058 France; 5 School of Pharmacy, Lebanese International University, Beirut, Lebanon; 6 Department of Biomedical Sciences, School of Arts and Sciences, Lebanese International University, Bekaa, Lebanon; 7 Center for Applied Mathematics and Bioinformatics (CAMB), Gulf University for Science and Technology (GUST), Hawally, Kuwait; 8 Social and Education Sciences Department, School of Arts and Sciences, Lebanese American University, Jbeil, Lebanon; 9 School of Medicine and Medical Sciences, Holy Spirit University of Kaslik, P.O. Box 446, Jounieh, Lebanon; 10 Psychology Department, College of Humanities, Effat University, Jeddah, 21478 Saudi Arabia; 11 Applied Science Research Center, Applied Science Private University, Amman, Jordan

**Keywords:** Climate change anxiety, Climate change, Psychometrics, Scale validation, Arabic

## Abstract

**Background:**

The Arab world is one of the global regions the most directly concerned by, and suffering from climate change’s adverse consequences. As such, there appears to be a strong need for an understanding of how Arab people may emotionally respond to climate change. Providing valid and reliable measures of climate change anxiety (CCA) can help gain a clear overview of the situation in Arab countries, and allow to intervene timely and effectively to mitigate any adverse effects on Arab people’s mental health. To this end, the present study sought to validate the Arabic language version of the Climate Change Anxiety Scale (CCAS) in a sample of native Arabic-speaking adults from the general population of Lebanon.

**Methods:**

This study adopted a cross-sectional approach and enrolled 763 adults between July and September 2023.

**Results:**

A confirmatory analysis of the one-factor model showed poor fit indices as follows: CFI = 0.90, GFI = 0.83, SRMR = 0.048 and RMSEA 0.131 [90% CI 0.123, 0.138). The two-factor model showed a satisfactory fit with a high CFI of and a GFI of 0.91 and a SRMR of 0.04 and RMSEA of 0.05 [90% CI 0.04, 0.06]. Both McDonald’s omega and Cronbach alpha values were high for the overall CCAS score (α = 0.96 and ω = 0.96) in the whole sample. Configural, metric and scalar invariance across gender was demonstrated. No significant difference was found between males and females in terms of total CCAS scores (24.53 ± 10.59 vs. 26.03 ± 11.17, t(761) = -1.82, *p* = .069). Higher CCA, functional impairment and cognitive impairment scores were significantly associated with higher depression, anxiety and stress.

**Conclusion:**

The reliability and validity of the CCAS in its Arabic version were proven. The availability of this self-report measure could offer a chance to assess CCA among Adults speaking Arabic, and to spread its future use for screening and research purposes.

## Introduction

Climate change has a negative influence on all forms of life on the globe, making it a major concern for the scientific community [1] and one of the biggest current challenges for human societies [2, 3]. Indeed, humans are shown to be largely affected, directly or indirectly, both physically and mentally, by devastating impacts of climate change, such as floods [4], hurricanes [5], loss of forests [6], fires [7], heat waves [8], rising temperatures [1], droughts [7], and tornadoes [9]. According to the Intergovernmental Panel on Climate Change’s report [10], the average global temperatures are anticipated to steadily climb, and extreme heat events are expected to become more common and severe over the next years, further posing considerable threats to human health. In particular, serious, extensive and cumulative climate change-related consequences on mental health have been predicted [11, 12]. For instance, a series of surveys documented that climate change is linked to a range of unpleasant negative emotions, including guilt, anger, helplessness [13], stress [14], sadness, hopelessness, fear [15], worries of being personally harmed [16], increased anxiety [13, 17], death anxiety and psychotic experiences [18]. It has, therefore, been claimed that the close connection between climate change and health consequences is a sufficiently solid reason why healthcare professionals need to actively participate in climate change preparedness, response and mitigation efforts [19].

It has been specifically emphasized that climate change anxiety (CCA) is one of the most prevalent and significant emotional responses experienced as a result of climate crisis [20, 21]. In addition, CCA has been found to engender significant daily life problems, and to even cause secondary mental health issues [17]. It is of note, however, that people are not similarly affected by worries about climate change. Those who are younger, more exposed to climate change-related media content, and/or living in geographically vulnerable areas seem to be more at risk of CCA [14, 22, 23]. Several definitions have been given to describe CCA. Some researchers have delineated it as “a generalized sense that the ecological foundations of existence are on the brink of collapsing” ([24], p. 250), or as “a chronic fear of a doomed environment” [25], whereas it has been regarded by others as “difficult feelings because of the ecological crisis” ([26], p. 14), as “apprehension and stress about anticipated threats to salient ecosystems” [27], or as an “anxiety which is significantly related to anthropogenic climate change” [26]. Overall, recent research highlights that CCA is multidimensional in nature [26, 28]. To help operationalize such a construct with heterogeneous definitions, accurately measure its severity, provide timely preventive action and allow cross-studies comparisons, Clayton and Karazsia [20] designed and validated the Climate Change Anxiety Scale (CCAS).

The CCAS assesses psychological responses to climate change. The scale is composed of 13 items scored on a five-point rating scale ranging from 1 (never) to 5 (almost always) and divided into two subscales [20]. The first subscale, i.e. cognitive and emotional impairment (items 1–8; e.g., “Thinking about climate change makes it difficult for me to concentrate”), reflects a range of responses to climate change, such as difficulty concentrating or sleeping, nightmares, crying, or rumination. The second subscale, i.e. functional impairment (items 9–13; e.g., “My concerns about climate change interfere with my ability to get work or school assignments done”) reflects the way how climate change may interfere with one’s capacity to socialize or work [20]. The CCAS was originally validated in a sample of US adults, demonstrating high internal consistency reliability (Cronbach’s alpha for all scales of > 0.80), good concurrent and discriminant validity in terms of its significant correlations with measures of depression, anxiety, negative emotionality and environmental identity (i.e., one’s emotional connection to, and perception of identification with nature). Other properties of the scale, such as cross-gender invariance [29] and test-retest reliability [30] were also demonstrated. Later, the CCAS has been adapted and validated in different countries into different languages, including Italian [30], German [31], French [32], Polish [29], Korean [33], Finnish [34], and Filipino [35]. Although these versions generally confirmed the adequate psychometric properties of the CCAS in different populations and backgrounds, there was some uncertainty regarding the factorial validity of the scale. For instance, Wullenkord et al. [31] were not able to replicate the originally proposed bidimensional factor model in the German language, and argued that the CCAS rather captures a single latent entity of climate-related emotional impairment through 12 items that loaded into two factors. In contrast, Mouguiama-Daouda et al. [32] found that the 13-item two-factor structure of the CCAS in its French version outperformed the one-factor structure. To date, no Arabic version of the CCAS is available to the best of our knowledge.

The Arab world is one of the global regions the most directly concerned by, and suffering from climate change’s adverse consequences [36]. The extreme climate events and increasing occurrence of natural disasters, coupled with the economic, political, and social challenges faced by Arab states continue to increase the susceptibility of the region [36]. All these effects are expected to magnify preexisting limited resources (such as poor access to healthcare), making Arab people even more vulnerable to deleterious climate change effects [37]. At the same time, however, and due to the multiple ongoing conflicts in the region, climate change has been largely underreported by local media in most Arab countries, and was rather covered as “foreign” (international) news [38]. In light of these data, there appears to be a strong need for an understanding of how Arab people may emotionally respond to climate change. Providing valid and reliable measures of CCA can help gain a clear overview of the situation in Arab countries, and allow to intervene timely and effectively to mitigate any adverse effects on Arab people’s mental health. To this end, the present study sought to validate the Arabic language version of the CCAS in a sample of native Arabic-speaking adults from the general population of Lebanon. As study hypotheses, we expect that confirmatory factor analysis will yield similar factor structure to the original version of the scale invariantly across gender groups, and that the Arabic CCAS will demonstrate adequate composite reliability, and good concurrent validity through significant positive correlations with measures of depression, anxiety and stress.

## Methods

### Study design and participants

This study adopted a cross-sectional approach involving adults aged 18 years and above. The data collection tool was developed in Arabic, Lebanon’s native language, and distributed via a snowball sampling method between July and September 2023. Ten university students, who had received training from the research team, were responsible for disseminating the survey through a Google Form link. They shared the link with their acquaintances, who in turn forwarded it to their family members and friends from all Lebanese districts (Beirut, Mount Lebanon, North, South, and Bekaa). Further promotion to the study took place on the social media. To be eligible for participation, individuals needed to be Lebanese residents and 18 years and above. Those who declined to complete the questionnaire were excluded. Participants were initially encouraged to seek parental consent, and once granted they provided digital informed consent. Subsequently, they were instructed to complete the questionnaire, which was presented in a pre-randomized order to control for any sequencing effects.

### Measures

#### Demographic characteristics

Participants were requested to provide their demographic information, which encompassed age, gender, education level, place of living (urban/rural) and household crowding index, reflecting the socioeconomic status of the family, calculated by dividing the number of persons by that of the rooms in the house except the kitchen and bathrooms.

#### Climate change anxiety scale (CCAS)

The Climate Change Anxiety Scale (CCAS) comprises 22 items designed to assess emotional reactions to climate change [20]. It encompasses four sub-scales, evaluating cognitive and emotional impairment, functional impairment, personal climate change experiences, and behavioral engagement. Respondents rate each item on a 5-point scale, ranging from “never” (1) to “almost always” (5). Total scores for each subscale and the overall CCAS are computed by summing responses, with higher scores indicating greater anxiety levels. The scale underwent translation into Arabic using a rigorous forward-backward method. An independent Lebanese translator, unrelated to the study, initially translated the English content into Arabic. The Arabic version was subsequently translated back into English by a proficient Lebanese psychologist with full proficiency in English. Any literal or context-specific translations were reconciled by the translation team. To ensure translation accuracy, a panel of experts, consisting of the research team, a psychologist, a psychiatrist, and the two translators, scrutinized both the original English versions and the translated versions, rectifying any disparities. A pilot study was done on 30 participants to make sure all questions are clear.

*Depression*,* Anxiety and Stress Scale-8 items (DASS-8)*: Validated in Arabic [39], it’s a self-report questionnaire used to measure psychological distress. This scale is composed of eight items, in three subscales: depression (three items including ‘’ felt down hearted and blue’’), anxiety (three items including: ‘’ felt scared without reason’’), stress (two items including: ‘’ was using a lot of my mental energy ‘’), rated on a 4 point Likert scale: ranging from ‘’0 = Did not apply to me at all’’ to ‘’3 = Applied to me very much or most of the time’’ [39], higher scores indicate more psychological distress (ω = 0.93 / α = 0.93).

### Statistical analysis

Confirmatory factor analysis (CFA) was conducted on the whole sample (*N* = 763) to test the original (one-factor) and the two-factor structures of the Climate Change Anxiety Scale (CCAS) [33]. The CFA was performed using RStudio (Version 1.4.1103 for Macintosh) [39, 40] and the Lavaan [41] and semTools packages [42]. The Weighted Least Squares with Mean and Variance (WLSMV) estimation method, which is known to be more appropriate for ordinal data [43], was used. The CFA model was considered to fit well if the Tucker–Lewis index (TLI) and the Comparative Fit Index (CFI) were > 0.95, and the Standardized Root Mean Square Residual (SRMR) was < 0.08 [44].

**Gender invariance.** To examine gender invariance of the The CCAS scores, we conducted multi-group CFA [45] using the total sample. Measurement invariance was assessed at the configural, metric, and scalar levels [46]. We accepted ΔCFI ≤ 0.010 and ΔSRMR ≤ 0.010 as evidence of invariance [45].

To assess the reliability of the scale, we computed McDonald’s omega and Cronbach’s alpha values, with a cutoff value of 0.7 and abov considered adequate. The total climate anxiety and functional impairment and cognitive impairment subscales scores were considered normally distributed as their skewness and kurtosis values varied between − 1 and + 1. The Student t test was used to compare the scores between genders, whereas the Pearson test was used to correlate continuous variables. *P* < .05 was deemed statistically significant.

## Results

### Description of the sample

Seven hundred sixty-three participants filled the survey, with a mean age of 28.57 ± 11.08 years. A total of 63.4% participants were females and 80.5% had a university education level. Other details are summarized in Table 1.

**Table 1:** Sociodemographic and other characteristics of the sample (*n* = 763)

Gender	
Males	279 (36.6%)
Females	484 (63.4%)
Education level	
Secondary or less	149 (19.5%)
University	614 (80.5%)
Place of living	
Urban	372 (48.8%)
Rural	391 (51.2%)
Age (in years)	28.57 ± 11.08
Household crowding index (person/room)	1.15 ± 0.52
Climate change anxiety	25.48 ± 10.98
Functional impairment	13.77 ± 6.06
Cognitive/emotional impairment	1172 ± 5.19
Stress	2.59 ± 1.74
Anxiety	3.43 ± 2.50
Depression	3.49 ± 2.48

### Confirmatory factor analysis

A confirmatory analysis of the one-factor model showed poor fit indices as follows: CFI = 0.90, GFI = 0.83, SRMR = 0.048 and RMSEA 0.131 [90% CI 0.123, 0.138).

A two-factor confirmatory analysis was conducted to test the factor structure of the CCAS found in the Korean validation study [33]. This model showed a satisfactory fit with a high CFI of and a GFI of 0.91 and a SRMR of 0.04 and RMSEA of 0.05 [90% CI 0.04, 0.06]. Factor loadings are can be found in Table 2. Both McDonald’s omega and Cronbach alpha values were high for the two subscales (cognitive/emotional impairment α = 0.91 and ω = 0.91, functional impairment α = 0.93 and ω = 0.93) and overall CCAS score (α = 0.96 and ω = 0.96) in the whole sample.

### Measurement invariance

This two-factor model was further used as the basis for assessing gender invariance for the scale. Model fit for configural, metric and scalar invariance is provided in Table 3. No significant difference was found between males and females in terms of total CCAS scores (24.53 ± 10.59 vs. 26.03 ± 11.17, t(761) = -1.82, *p* = .069), functional impairment (13.27 ± 6.00 vs. 14.05 ± 6.08, t(761) = -1.73, *p* = .085) and cognitive impairment (11.26 ± 4.90 vs. 11.98 ± 5.33, t(761) = -1.88, *p* = .060).

**Table 2:** Standardized loading factors of the climate anxiety scale deriving from the Confirmatory Factor Analysis

	Factor 1 = Cognitive/emotional impairment	Factor 2 = Functional impairment
1. Thinking about climate change makes it difficult for me to concentrate.	0.74	
2. Thinking about climate change makes it difficult for me to sleep.		0.75
3. I have nightmares about climate change.		0.87
4. I find myself crying because of climate change.	0.88	
5. I think, “why can’t I handle climate change better?”	0.87	
6. I go away by myself and think about why I feel this way about climate change.	0.89	
7. I write down my thoughts about climate change and analyze them.	0.89	
8. I think, “why do I react to climate change this way?”	0.88	
9. My concerns about climate change make it hard for me to have fun with my family or friends.		0.89
10. I have problems balancing my concerns about sustainability with the needs of my family.		0.83
11. My concerns about climate change interfere with my ability to get work or school assignments done.		0.90
12. My concerns about climate change undermine my ability to work to my potential.		0.91
13. My friends say I think about climate change too much.		0.89

**Table 3:** Fit indices of the two-factor model confirmatory factor analysis and the measurement invariance across genders

	Robust TLI	Robust CFI	SRMR	SBχ²	df	Model Comparison	ΔRobust CFI	ΔRobust TLI	ΔSBχ²	Δdf	Pr(> Chisq)
Original model	0.953	0.961	0.046	594.198	64						
Config-model	0.966	0.967	0.052	167.15	150	Configural vs. metric	0.003	0.002	13.034	11	0.2911
Metric-model	0.968	0.964	0.051	162.02	139	Metric vs. scalar	0.003	0.002	13.034	11	0.2911
Scalar-model	0.966	0.967	0.052	167.15	150						

*Note.* CFI = Comparative Fit Index; RMSEA = Steiger-Lind Root Mean Square Error of Approximation; SRMR = Standardized Root Mean Square Residual; SBχ² = Satorra-Bentler Chi-square.

### Construct validity

Higher climate anxiety, functional impairment and cognitive/emotional impairment scores were significantly associated with higher depression, anxiety and stress scores (Table 4).

**Table 4:** Correlation matrix of continuous variables

	1	2	3	4	5	6
1. Climate anxiety	1					
2. Functional impairment	0.98***	1				
3. Cognitive/emotional impairment	0.97***	0.91***	1			
4. Stress	0.24***	0.25***	0.23***	1		
5. Anxiety	0.37***	0.37***	0.35***	0.77***	1	
6. Depression	0.35***	0.36***	0.32***	0.77***	0.84***	1

## Discussion

Arab countries are amongst the most impacted by climate change worldwide, both environmentally and economically, with a considerable lack of adaptive resources and mitigation strategies [47]. There is increasingly overwhelming evidence that climate change represents “the biggest threat to global mental health in the coming century” [48]. Therefore, tackling this threat should be a priority for all mental healthcare providers, in particular those from vulnerable regions of the world such as the Middle East and North Africa. In this perspective, this study proposed to make available a psychometrically reliable instrument for sound assessment of emotional response to climate change among Arabic-speaking individuals. The CCAS was translated to the Arabic language and its psychometric properties were examined. Findings supported a two-factor solution with all 13 items, good reliability, and appropriate concurrent validity. Gender invariance was also established. Overall, findings suggest that the Arabic CCAS is suitable for use as a self-report measure of CCA among Arabic-speaking adults.

Confirmatory factor analysis with 763 young adults from the general population supported the two-dimensional model suggested by the original developers of the scale [20], with one factor reflecting the cognitive/emotional impairment dimension and the other factor the functional impairment dimension. A slight difference was found with the original validation, as two items about sleep problems (item 2 “Thinking about climate change makes it difficult for me to sleep” and item 3 “I have nightmares about climate change”) loaded into Factor 2 (i.e. Functional impairment). The one-factor model was also tested, but showed poor fit indices. It should be emphasized that the Clayton and Karazsia [20] did not assess the factor structure of the 13-item CCAS. Subsequent validation studies showed conflicting findings regarding the factorial validity of the CCAS, with previous studies demonstrating either a two-factor structure (with 13 items [20, 30, 32, 35]), a single-factor structure (with 12 items [31]; with 10 items [34]), or a three-factor structure (i.e., intrusive symptoms, functional impairment, and reflections on climate anxiety; [29]). In line with our findings, the Korean version of the CCAS obtained a two-factor structure with 13 items, albeit with a slightly different distribution of items [33]. The differences observed in factor structure across previous psychometric studies may be explained by the cultural variations in CCA. Altogether, most of the previous linguistic validation studies conducted in different countries and cultural backgrounds concur with the multi-dimensional nature of the CCA experience in healthy adults. Based on the large variations in factor solutions, some authors recommended to use the overall CCAS latent score in cross-cultural research [29]. further research is clearly warranted to explore and confirm the factor structure of the CCA construct.

Both McDonald’s omega and Cronbach alpha values were excellent for the two subscales and the overall CCAS score in the whole sample, thus indicating good scale reliability. Consistent with these results, all previous validation studies on healthy samples (from Italy [30], Germany [31], France [32], Poland [29], Korea [33], Philippine [35]), including the initial validation [20], have provided empirical support that the CCAS has satisfactory internal consistency reliability. The present study is among the very few studies (e.g., [29]) that have tested and established measurement invariance across gender, which signifies that the same latent variable structure is being assessed between male and female respondents. This is a relevant finding, given that emotional response to climate change may vary across male and female adults. However, sound gender comparisons can only be made if the CCA construct and the CCAS items have the same meaning across gender groups. Finally, the present results showed that the construct validity of the Arabic version of the CCAS was established against measures of depression, anxiety and stress. These findings support those of previous studies on the significant positive correlations between climate anxiety (as assessed using the CCAS) and anxiety-depressive symptoms (e.g., [20, 29, 32]), thus providing further evidence that CCA is a psychological response closely connected to negative emotions and experiences.

### Limitations

This study has some limitations that need to be discussed before any conclusions can be drawn. First, an online approach was adopted, resulting in a majority of female, highly educated respondents, which may limit the representativeness of our sample to the whole Lebanese adult population. Second, the study involved Arabic-speaking participants from one Arab country and culture (i.e., Lebanon), whereas those residing in other Arab states could be exposed in a different manner to climate change impacts in their environments, and/or may perceive and respond to climate change differently. It is, therefore, highly recommended that the psychometric properties of the Arabic CCAS be verified in other geographical and sociopolitical contexts. Finally, other psychometric qualities of the Arabic CCAS still need to be examined in future research, such as test-retest reliability.

## Conclusion

In sum, findings revealed that the reliability and validity of the CCAS in its Arabic version were proved. The availability of this self-report measure could offer a chance to assess CCA among Adults speaking Arabic, and to spread its future use for screening and research purposes. This can, in turn, help shed light on the growing threat of climate change’s devastating repercussions in Arab countries, where the issue has until recently been neglected in public policy debates, as well as in healthcare professional practice and research. In particular, the Arabic CCAS may offer a unique opportunity to conduct large epidemiological studies to generate evidence on the extent to which Arabic-speaking general population adults are affected by CCA. This will enable to draw a broad overview of the situation in Arab countries to lead key government decision-makers toward recognizing the necessity of considering climate change effects on the population’s mental health in decision-making and policy. This can also improve Arab young people’s understanding of the climate crisis, prevent or manage their emotional reactions to it, break the “climate silence”, and help reconcile individual and political actions against climate change.

## Data Availability

No datasets were generated or analysed during the current study.
